# Changes of Migration, Immunoregulation and Osteogenic Differentiation of Mesenchymal Stem Cells in Different Stages of Inflammation

**DOI:** 10.7150/ijms.58428

**Published:** 2022-01-01

**Authors:** Feng Gu, Ke Zhang, Jiangbi Li, Xiaoping Xie, Qiangqiang Wen, Zhenjiang Sui, Zilong Su, Tiecheng Yu

**Affiliations:** Department of Orthopedics, First Hospital of Jilin University, Changchun 130021, Jilin, China.

**Keywords:** Mesenchymal Stem Cells, Infection, Immunomodulatory, Differentiation

## Abstract

Bone infection has always been the focus of orthopedic research. Mesenchymal stem cells (MSCs) are the natural progenitors of osteoblasts, and the process of osteogenesis is triggered in response to different signals from the extracellular matrix. MSCs exert important functions including secretion and immune regulation and also play a key role in bone regeneration. The biological behavior of MSCs in acute and chronic inflammation, especially the transformation between acute inflammation and chronic inflammation, has aroused great interest among researchers. This paper reviews the recent literature and summarizes the behavior and biological characteristics of MSCs in acute and chronic inflammation to stimulate further research on MSCs and treatment of bone diseases.

## Introduction

Bone infection has always been the focus of orthopedic research. Bone infection is a serious complication in orthopedics and often leads to severe bone destruction and nonunion. When acute infection is not effectively controlled, it may become chronic, which often leads to disastrous consequences. Osteomyelitis is a typical bone infection, and it often requires prolonged antibiotic treatment and surgical intervention. Osteomyelitis is caused by bacterial pathogens, and *Staphylococcus aureus* and *Staphylococcus epidermidis* are responsible in most cases 1. Mesenchymal stem cells (MSCs) originate from the mesoderm and are characterized by the potential of self-proliferation and multi-directional differentiation, and can differentiate into osteoblasts, chondrocytes, and adipocytes *in vitro* 2-4. The potential of MSCs to differentiate into different tissue types has made the study of MSCs a hot research topic in recent years. MSCs are the natural progenitors of osteoblasts and are involved in the process of osteogenesis in response to different signals from the extracellular matrix (ECM), and play a critical role in the bone regeneration and immunity 5-7. As a result, the study of the response of MSCs to inflammation and its different stages are of great interest.

During the process of bone regeneration, inflammatory factors can activate and mobilize endogenous MSCs, which migrate from their niche to the damaged site 8. MSCs can promote the regeneration of bone tissue through osteogenic differentiation and are the key cells involved in fracture healing. However, infection often leads to progressive bone destruction and loss, and eventually bone nonunion occurs when inflammation persists and becomes chronic. Bone defects and nonunion caused by infection have always been difficult to treat clinically, not only because inflammation is difficult to control, but also because the differentiation and osteogenic ability of MSCs and osteoblasts is altered under a chronic state of inflammation. Acute and chronic inflammation are often associated with pathogens, and the interplay between infection and inflammation is of paramount importance to clinical outcomes 9. Therefore, the migration, differentiation, immune regulation, secretion, and other functions of MSCs in acute and chronic inflammation have become an area worthy of study. Following a review of the literature, we summarized the biological performance of MSCs in acute and chronic inflammation. In infection and inflammation MSCs interact with immune cells to intricately regulate cellular activity. Differences in the behaviors of MSCs in acute and chronic inflammations have also become the focus of significant research attention. It is known that the transformation from acute inflammation to chronic inflammation is a key feature of tissue repair. The behaviors of MSCs in the acute and the chronic inflammations also suggest that if the mechanisms involved in the transformation between the two stages are better understood, intervention on MSCs activities in the infection would be made possible, and the basis for treating the bone infection be provided.

## Inclusion of references

A literature search was conducted with PubMed, EMBASE and Web of Science databases. The following terms: ('mesenchymal stem cells', 'acute inflammation', 'chronic inflammation', 'migration', immunoregulation' and 'osteogenic'), were used to search eligible studies from 2002 to 2020, with 80 relevant English articles included in this review.

## Signal pathway related to osteogenesis of MSCs

It is know from previous studies that in the process of regulating the osteogenic differentiation of MSCs, signaling pathways such as mitogen-activated protein kinases (MAPK) 10, bone morphogenetic protein (BMP)/mothers against decapentaplegic homolog (SMAD) 11-13, Wnt/β-catenin 14,15 and PI3K-Akt-Mtor 16 play a key role.

MAPK is a conserved family of enzymes that can transmit and spread external stimuli, and coordinate cell responses through phosphorylation cascades to adapt to environmental changes 10,17. It is a proline-directed serine/threonine (Ser/Thr) specific protein kinase that regulates cell activities, such as gene expression, mitosis, differentiation, and cell survival/apoptosis. So far, four different types of mammalian MAPKs have been identified as extracellular regulated protein kinases 1 and 2 (ERK1 and 2), c-Jun N-terminal kinases 1-3 (JNK 1-3), p38MAPK (p38α, p38 β, p38γ and p38δ) and ERK5 18. Activated ERK, JNK and p38MAPK have different signal pathways, but there is also cross-regulation between the signal pathways. p38MAPK contains a set of 4 proteins, i.e., p38α, p38β, p38γ and p38δ, which represent different branches of the phylogenetic tree and are each conserved in species. Among the p38MAPK family, p38α is the most widely expressed and studied; p38β and δ are expressed in immune cells, and p38γ is a highly restricted expression which has not been fully elucidated yet 18. Phosphorylated p38MAPK can activate activator protein 1 (AP-1), transcription factor 1 (SP-1) and NF-κB and other signaling pathways, and promote the secretion of various cytokines and inflammatory mediators. In the so-called classical p38MAPK signaling pathway, phosphorylation of p38MAPK in the cytoplasm can activate several transcription factors, including AP-1 and SP-1, regulate their movement to the nucleus, and then transcribe several inflammatory mediators and cytokines 18. The AP-1 components, mainly members of the Fos family such as c-Fos and Fos related antigen-1 (Fra-1), have important functions in both of these cell types 19.

In addition, the phosphorylated p38MAPK activates mitogen and stress-activated protein kinases 1 (MSK 1) and mitogen and stress-activated protein kinases 2 (MSK 2), which can phosphorylate the NF-κB complex transactivating Ser276 of the p65 subunit, thereby enhancing the NF-κB signaling 18. The phosphorylated AP-1, SP-1 and NF-κB increase the secretion and synthesis of a variety of inflammatory cytokines, and the body's immune defense system is thus activated 18.

BMPs are members of the transforming growth factor β (TGF-β) superfamily 20. They are powerful osteogenic differentiation factors in the process of bone formation and play an important regulatory role therein. Intracellular BMP signal transduction is mainly mediated by the SMAD protein. The SMAD protein binds to transmembrane receptor-threonine kinase through BMP and gets activated 20. The activated SMADs are then transported to the nucleus, where they regulate the expression of genes involved in osteogenesis. BMP2 can induce bone formation and differentiation *in vivo* and *in vitro*. It activates SMAD1/5/8 and several mitogen-activated protein kinases (MAPK), which ultimately leads to the increase in the runt-related transcription factor 2 (RUNX2) and the core binding factor A1 (CBFA1) 20.

The SMAD protein family can be divided into 3 categories, the receptor-activated SMAD (R-SMAD), the common pathway SMAD (Co-SMAD) and the inhibitory SMAD (I-SMAD) 13. R-SMAD can be activated by type I receptors and form transient complexes of receptors. It is further divided into two categories, the AR-SMAD activated by activin TGF-β, including SMAD 2, SMAD 3; and BMP. Other activated BR-SMAD includes SMAD 1, SMAD 5, SMAD 8, and SMAD 9 30. Co-SMAD includes SMAD 4, which is a common mediator required in various signal transduction processes of the TGF-β family 13. I-SMAD includes SMAD 6 and SMAD 7, which can bind to activated type I receptors to inhibit or regulate the signal transduction of the TGF-β family 21. The SMAD protein family plays a key role in the process of transmitting TGF-β signals from cell surface receptors to the nucleus, and different SMADs mediate the signal transduction of different TGF-β family members 13. The receptor complex formed by TGF-β as a ligand activates SMAD to enter the nucleus and jointly activate or inhibit the transcription of target genes regulated by them 21. PI3K-Akt-mTOR signaling pathway regulates many key functions of cells 22. Intracellular signaling mediates phosphoinositide 3-kinase (PI3K), Akt (protein kinase B/PKB) and mammalian target of rapamycin (mTOR) to form a signal network 22. The members of this signaling network control the expression of proteins that regulate apoptosis and cell cycle of progression/proliferation. They are very important for cell transportation/migration, and are also important regulators of cell metabolism and differentiation.

The Wnt/β-catenin signaling pathway regulates the osteogenic differentiation, bone formation and bone metabolism disorders of bone marrow mesenchymal stem cells 15,23. The activation of this typical pathway depends on the binding of Wnt, frizzled receptors and LRP co-receptors to the cell membrane, which will inhibit the expression of glycogen synthase kinase-3b (GSK3b) and promote the β-catenin migration to the nucleus 23. In the nucleus, the β-catenin enhances the transcription regulated by TCF4 (T-cell factor 4)/LEF1 (lymphoid enhancing factor-1), and the β-catenin-TCF4 complex activates the downstream targets gene transcription 24.

## MSCs in the stage of acute inflammation

### Migration ability of MSCs in acute inflammation

MSCs will recruit and exert biological effects under the action of cytokines and chemokines in acute inflammation, and this process also allows the organism to heal from infections and tissue damage. Infection or trauma to tissues is usually detected by receptors largely located on the surface of innate immune cells, which are called pattern recognition receptors (PRRs). These PRRs then identify the pathogen-related molecular patterns or damage-associated molecular patterns and trigger the inflammatory response. Acute inflammation peaks at 24 to 48 h after injury and is complete after about 7 days 25-27. MSCs undergo a process of selective migration to the site of injury and continuously secrete various nutritional factors, a function called “homing” 28. MSCs are then selectively recruited to the damaged or inflammatory tissues, based on the release of multiple inflammatory factors by the injured tissues 29. This process is essential for healing of the infection and bone regeneration. The migration ability of MSCs is increased in the acute inflammation, a process related to the release of tumor necrosis factor-α (TNF-α) and the activation of the NF-κB pathway 30,31. *Ward et al.* suggested that the up-regulation of podoplanin in the infected state is associated with the increased migration ability of MSCs 32. Toll-like receptors (TLRs) are a family of PRRs and are responsible for identifying the exogenous pathogens. TLRs are mainly expressed by first-line immune cells, such as neutrophils, macrophages, and dendritic cells (DCs). These receptors, which belong to the innate immune pathway, can recognize different microbial structures and induce various immune responses to specific pathogens. TLRs are expressed not only by immune cells, but also by MSCs. MSCs directly recognize pathogens through the TLRs receptors expressed on the cell surface and regulate the biological response of the cells to the acute inflammation. TLRs are activated by their respective endogenous ligands; for example, TLR-2 recognizes lipoteichoic acid (LTA), TLR-4 recognizes lipopolysaccharide (LPS), and TLR-5 recognizes flagellin, which leads to the activation of MSCs and to the generation and release of cytokines and chemokines 28,33. The activation of TLRs may affect the migratory function of MSCs. The TLRs expressed by MSCs from different sources may differ 33, and the activation of TLRs may not always play a positive role in the migration of MSCs. Although, in most cases, TLRs will enhance the migration of MSCs, there are studies indicating that TLR-3 inhibit migration 34,35. The change of migration status of MSCs in infection is complex, and the function of TLRs is worthy of further study to understand the underlying mechanisms.

### Osteogenesis differentiation of MSCs in acute inflammation

**Figure 1:**
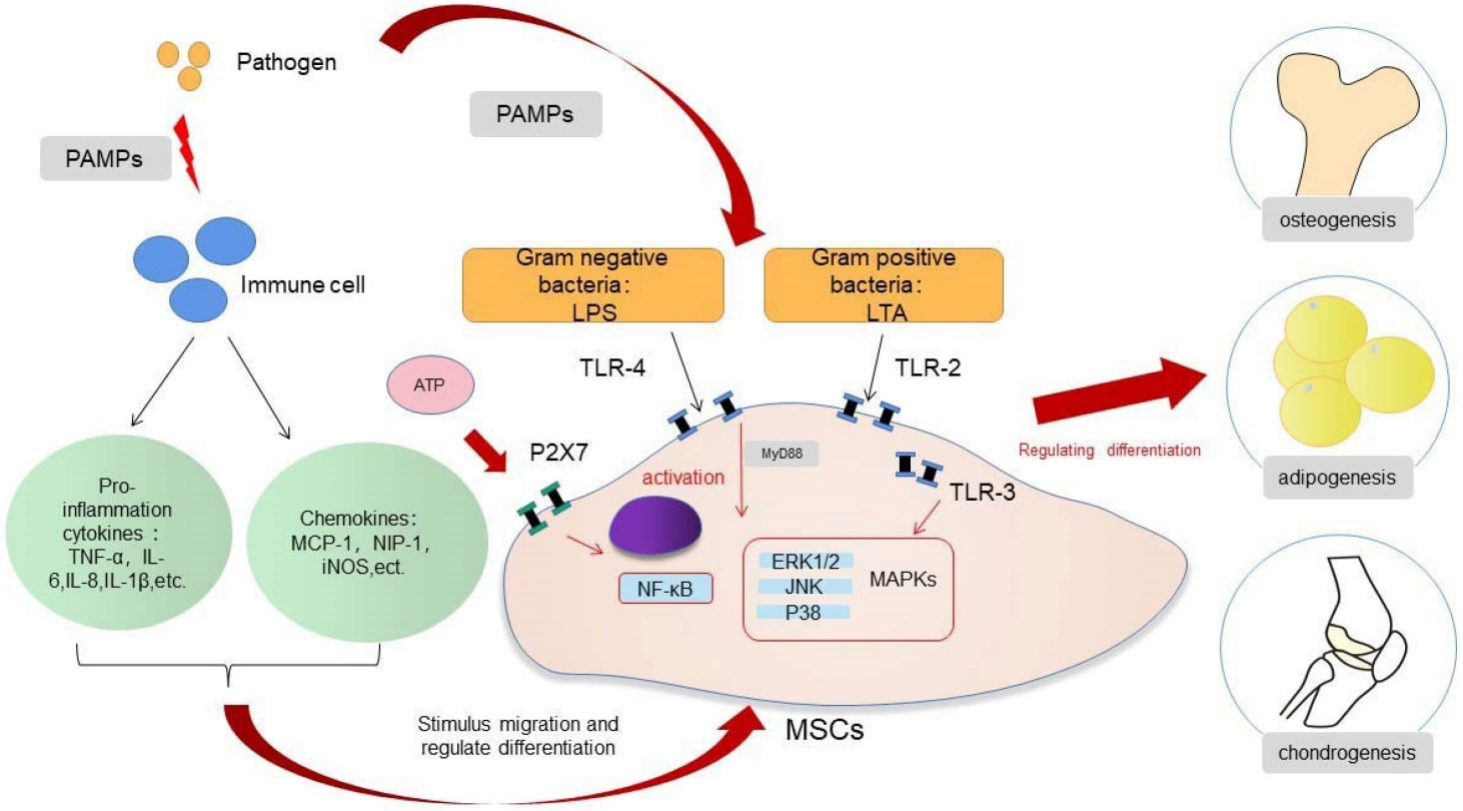
Regulation of MSCs differentiation in inflammation.

In the presence of acute infection, the acute inflammation response plays an important role in bone regeneration, and it is a crucial first step for bone healing. By contrast, the absence and inhibition of an acute inflammatory response may lead to impaired bone healing 27. The outcome of an appropriate acute inflammation response in the early stages is favorable to osteogenesis, although the response may also switch to a negative regulatory effect when “over-stimulated”. Previous studies have shown that drugs such as non-steroidal anti-inflammatory drugs (NSAIDs), chemotherapeutic agents and corticosteroids may increase the risk for nonunion 36,37. However, an excessive acute inflammatory response will increase the risk for impaired bone regeneration 38. The specific regulatory mechanisms involved in the acute inflammatory response on osteogenic differentiation warrants more in-depth study. If the extent to which an “appropriate” inflammatory response could be determined, then the mechanism of bone healing in the state of infection and trauma would be more clearly defined. The osteogenic differentiation of MSCs is associated with multiple pathways, and the mechanism involved is very complex (Fig. 1). When the bone is infected, the inflammatory response can regulate the osteogenic differentiation of MSCs in different ways. The main feature of acute infection is the release of proinflammatory immune factors. We found that the differentiation of MSCs in acute inflammation may proceed in different directions and induce both promotion and inhibition of bone formation. Under certain conditions, the inflammatory response will enhance the osteogenesis of MSCs. For example, studies have shown that low-dose Staphylococcus aureus exotoxin stimulation will enhance the osteogenesis of MSCs, and LTA stimulation will also promote the bone formation of MSCs 39,40. However, when the inflammatory response continues to increase, the osteogenic differentiation of MSCs begins to weaken, and apoptosis is increased. We know that proinflammatory factors will activate the NF-κB signaling pathway in acute inflammation, and activation of this pathway will promote β-catenin degradation and inhibit osteogenic differentiation 41. Several studies 42-44 have shown that the activation of the NF-κB pathway has an adverse effect on the osteogenic differentiation of MSCs 42-44. *Xu et al*. showed that the purinergic receptor p2x and ligand gated ion channel, 7 (P2X7) receptors are also key molecules that regulate the osteogenesis of MSCs in acute inflammatory conditions 45. P2X7 is a receptor located on the surface of cell membranes, which can be activated by adenosine triphosphate (ATP) and then can activate downstream MAPK receptors 46. The MAPK pathway is mainly divided into three cascades: the ERK 1/2, p38 MAPK, and c-Jun N-terminal kinase (JNK) pathways. Although there are differences in cellular function, all MAPKs signal through a similar mechanism of associated proteins and activate common downstream transcription factors, known as AP-1, RUNX2, and recombinant osterix (OSX) 47. Previous research reported here suggest that P2X7 receptor agonist directs differentiation toward the osteoblast lineage and away from the adipocyte lineage in MSCs by stimulating ERK1/2 and JNK signaling pathways in a P2X7R dependentway 46. P2X7R contributes to homeostatic control of MAPK cascades and shape the outcome of ERK signaling. P2X7R participates in feedforward loops to prolong ERK activation towards its cytoplasmic substrates 48,49. In the acute inflammatory state, expression of the P2X7 receptor will be significantly reduced and thus will reduce the osteogenic differentiation of MSCs 45,46.

### Osteogenesis differentiation of MSCs in heterotopic ossification

In the process of inflammatory response, the same cytokine will have different regulatory effects on the osteogenic differentiation of MSCs under different concentration and action time conditions. Studies have shown that low concentration of TNF-α (1 ng/ml) promotes the osteogenic differentiation of MSCs in the short term (1 d and 3 d) and increases the mRNA expression of BMP-2 and SMAD 1, but inhibits the osteogenic differentiation of MSCs at long term (7 d). However, whether long-term or short-term, high concentrations of TNF-α inhibited the osteogenic differentiation of MSCs and the expression of SMAD 1, but resulted in high expression of BMP-2 50.

Very similarly, studies have shown that IL-10 has concentration-dependent, dual roles in the osteogenesis of MSCs through P38MAPK and NF-kB signaling pathways. Under low-dose conditions (0.1-1 ng/ml), it can promote osteogenesis. But when the concentration of IL-10 exceeds 10 ng/ml, it will inhibit osteogenesis 51. Such experimental results are also consistent with the different manifestations of MSC's osteogenic ability in both acute inflammation and chronic inflammation.

Among the ossification caused by inflammation, heterotopic ossification is a very special type of decease, which we will separately describe in detail. Heterotopic ossification (HO) often occurs after severe trauma or burn 52 and the process during which HO forms is governed by MSCs that are attempting to regenerate tissue based on cues from the inflammatory niche 53. Heterotopic ossification often occurs primarily through endochondral ossification, which shows its common feature 53. *Evans et al* included 24 cases of 36 high-energy penetrating injuries, of which 13 (36%) had heterotopic ossification. After debridement, the tissue fragments and local exudate were tested for inflammatory factors and found that IL-6, IL-10 and monocyte chemoattractant protein-1 are significantly related to the occurrence of the heterotopic ossification 54. Transforming growth factor-β1 (TGF-β1) is also known as a critical regulator in this process 55,56. Formation of HO has been shown to require the recruitment of macrophages and mast cells for genetic and neurogenic HO 57-59. Previous study revealed predetermination of aberrant chondrogenic cell fate of the mesenchymal progenitor cell population as early as 3 days after injury, indicating that the inflammatory phase is the critical time window for therapeutic targeting 53. Although the formation process of heterotopic ossification is relatively slow, its mechanism does not seem to be the result of prolonged inflammatory response stimulation, but rather the gradual evolution of the inflammatory response after the acute trauma. MSCs were recruited to the injury site, and the environmental niche consisted of other cell types and microenvironmental factors are playing critical roles in influencing the fate of MSCs 60. Therefore, the occurrence of the heterotopic ossification is related to the inflammatory response, but its outcome is not a simple regulation of the acute inflammation or chronic inflammation, rather, it is caused by more complex reasons. The specific mechanism still needs further research and discussion.

### Immunomodulatory properties of MSCs in acute inflammation

**Figure 2:**
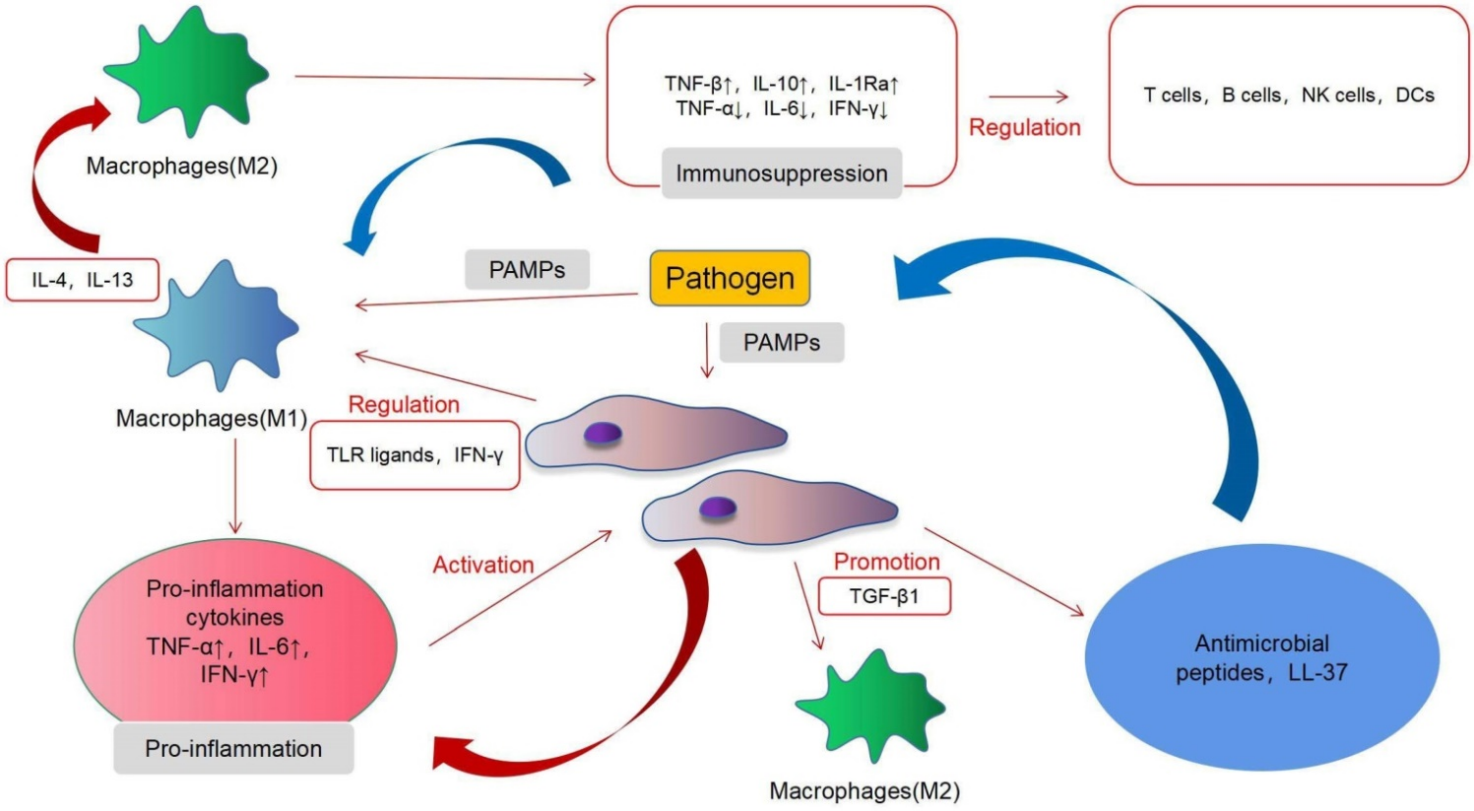
Immunomodulatory properties of MSCs in inflammation.

In acute infections, MSCs mainly exert immunosuppressive effects and, under certain conditions, may also exhibit proinflammatory effects. MSCs induce a complex regulation of a variety of immune cells in acute infection and interact with immune cells through proinflammatory and immunosuppressive regulatory directions. MSCs have immunomodulatory functions, which are mainly achieved by interacting with different immune cell subpopulations 61. T cells, B cells, natural killer (NK) cells, macrophages, dendritic cells (DCs) and other immune cells play an important role in our body, and MSCs can regulate their function (Fig. 2). MSCs mainly exert an immunosuppressive role in acute inflammation. T cells can be divided into helper T (Th) cells, cytotoxic T lymphocyte (CTL), and regulatory T cells (Tregs), according to their functions. Th cells can be divided into Th1, Th2, and Th17. Th1 can produce interleukin-2 (IL-2), interferon-γ (IFN-γ), transforming growth factor-β (TNF-β), and other immune factors, thereby activating macrophages and enhancing cellular immunity. Th2 can produce IL-4, IL-6, IL-10, and other products and activates B cells to enhance the humoral immunity. Tregs are a type of T cell with immunosuppressive effects 62. The main features of the T cell response involve cell proliferation and cytokine secretion. Inhibition of the proliferation is the most significant effect of MSCs on T cells. *In vitro*, MSCs can suppress the proliferation of T cells induced by mitogens and alloantigens. Although the exact mechanisms underlying the immunosuppressive effects of MSCs are still unclear, most evidence indicates that soluble factors are involved. These factors include prostaglandin E2 (PGE2), indoleamine 2,3-dioxygenase (IDO), hepatocyte growth factor (HGF), IFN-γ, and TGF-β1. MSCs can also alter the balance of Th1/Th2 cells 63 and T cell differentiation into Treg cells and downstream effects on acute inflammation and immune activity 64. MSCs can inhibit the differentiation, proliferation, activation, and chemotaxis of B cells under the action of the C-C motif ligand 2 (CCL2) and PGE2, but under the influence of vascular endothelial growth factor (VEGF), they will promote the differentiation, proliferation, activation, and chemotaxis of B cells 65. In acute inflammation, MSCs can mediate the transformation of M1 macrophages to the M2 type 66. Extracellular vesicles of MSCs also regulate the activation of B cells 67. In this process, the proinflammatory response mediated by TLR-4 and the immunosuppressive effect mediated by TLR-3 produce a bidirectional regulation of B cells and T cells 28. In acute inflammation, MSCs can mediate the transformation of M1 macrophages to the M2 type 68. M1 macrophages exert a proinflammatory effect and can secrete various cytokines and chemokines, whereas M2 macrophages are anti-inflammatory cells that induce immune tolerance and promote cell repair and regulation. A recent study has shown that murine MSCs shift macrophages to an M2 phenotype through TGF-β1 69. MSCs exerted this effect by inhibiting NF-κB p65 and by activating the signal transducers and activators of the transcription 3 (STAT3) pathways 70. Interfering with the conversion of M1 to M2 macrophages may become a breakthrough for the treatment of infectious diseases in the future. The effects of MSCs are not always immunosuppressive; however, MSCs can adopt a proinflammatory state under certain conditions. For example, the presence of low levels of IFN-γ and TNF can endow MSCs with immunostimulatory potential 71. MSCs produce PGE2 under inflammatory conditions, and factors such as IL-10 can inhibit the maturation of DCs and produce an immunosuppressive effect 64. Macrophage colony stimulating factor (M-CSF) inhibits the activation, differentiation, migration, endocytosis, and maturation of DCs during inflammation 72. Only a few studies have investigated the interaction of NK cells with MSCs. In their pioneering work, *Moretta et al*. showed that resting unstimulated MSCs are capable of inhibiting NK cell proliferation, cytokine release, and cytotoxicity, via PGE2 and IDO 73. More recently, studies have shown that although MSCs activated in an inflammatory state have a limited effect on NK cells, they can promote their differentiation 74.

## MSCs involvement in the stage of chronic inflammation

In chronic inflammation, the immune regulatory function and osteogenic differentiation ability of MSCs are altered, which eventually leads to the persistence of inflammation, bone destruction, and nonunion. The inflammatory response is an essential and critical step in the healing process of acute bone infections; however, when the pathogen is difficult to remove, it will turn into chronic infection. *Munir* et al. believe that in chronic inflammation, MSCs residing in tissues will acquire a proinflammatory phenotype opposite to that of normal MSCs and will then produce proinflammatory factors 75. This response can, to some extent, explain the nonunion and osteolysis observed in chronic osteomyelitis. In this process, bone tissue regeneration is accompanied by the repair of surrounding soft tissue damage. But in many cases, when the infection cannot be effectively controlled, the persistent inflammatory reaction will change from acute to chronic. In chronic inflammation, TNF-α and the NF-κB signaling pathways are continuously activated, which will induce the differentiation and activation of osteoclasts 27. An acute inflammation that persists may shift into a chronic inflammatory state and persevere for a prolonged period. The continuous expression of the NF-κB pathway can reduce the osteogenesis and migration of MSCs and the apoptosis of MSCs 34,42. M1 macrophage activation continuously produces cytokines, resulting in an imbalance of M1/M2 macrophages in chronic inflammation 76. Bacteria adapt to and persist within chronic wounds as a biofilm, a sign of chronic bacterial infection. The presence of a biofilm will continue to release soluble factors. These soluble factors will induce further proliferation and differentially affect the differentiation of MSCs, including osteogenesis and adipogenesis, and induce the apoptosis of MSCs 34.

## MSC as a novel approach for infection treatment

Because of the effective differentiation potential of MSCs and their immunoregulatory properties during the inflammatory response, MSCs have become highly regarded in tissue engineering, wound repair, immunotherapy, and infection treatment. Many studies have proposed MSCs as a treatment for infection.* Saeedi et al*. used MSCs primed by modified LPS to treat sepsis, and, in this context, MSCs presented strong antibacterial properties 77. *Yuan et al*. used MSCs to reduce methicillin-resistant Staphylococcus aureus infection in rat models 78. It is related to its immunomodulatory ability and to the production of antibacterial peptides LL-37 after stimulating MSCs 79. The immune regulatory ability of MSCs makes it possible to regulate various immune cells in the body under the infection state and enhance their antibacterial ability. Alternatively, the production of antibacterial peptides is also an important factor for the antibacterial ability of MSCs. Under the activation of TLR-2 and TLR-4 receptors, MSCs can produce antibacterial peptides, which can kill pathogens directly. Simultaneously, MSCs can also affect immune regulation. Through the interaction with immune cells in the body, the bactericidal effects are enhanced to achieve the purpose of treating infection. However, MSCs are immature to treat infections, and a prior study has shown that when MSCs are implanted into non-sterile bone defects, their overall trophic activity may enhance an infection and/or exacerbate osteomyelitis, which might also be associated with its immunosuppressive effects 80. Although the specific mechanisms are still not completely understood, the above-mentioned undesired effects undoubtedly suggest that MSCs are still associated with significant controversy and uncertainty as a conventional infection treatment method.

## Conclusion

In an acute inflammation response, MSCs will first migrate to the site of infection and then regulate their differentiation and immunological effects via the stimulation of inflammatory factors. When an acute infection shifts to a chronic inflammation, the immune regulatory function and osteogenic differentiation ability of MSCs will be changed, which will eventually lead to the persistence of inflammation, bone destruction, and nonunion. The differentiation and immune regulation ability of MSCs under infection is bidirectionally regulated through multiple pathways. The transformation between acute inflammation and chronic inflammation has become a crucial point of tissue repair. The behavior of MSCs in acute and chronic inflammation states is worthy of further study, which may reveal the key involvement of MSCs in the whole process of infection.

## Abbreviations

MSCs: mesenchymal stem cells
ECM: extracellular matrix
TNF-α: tumor necrosis factor-α
TGF-β: transforming growth factor-β
BMP: bone morphogenetic protein
Ser: serine
Thr: threonine
MSK 1: mitogen and stress-activated protein kinases 1
MSK 2: mitogen and stress-activated protein kinases 2
CBFA1: core binding factor factor A1
PI3K: phosphoinositide 3-kinase
PKB: protein kinase B
mTOR: mammalian target of rapamycin
GSK3b: glycogen synthase kinase-3b
TCF4: T-cell factor 4
LEF1: lymphoid enhancing factor-1
NF-κB: nuclear factor kappa-B
TLRs: Toll-like receptors
PRRs: pattern recognition receptors
PAMPs: pathogen-associated molecular pattern
DAMPs: damage-associated molecular patterns
LTA: lipoteichoic acid
LPS: lipopolysaccharide
ATP: adenosine triphosphate
NSAIDs: non-steroidal anti-inflammatory drugs
MAPK: mitogen-activated protein kinase
ERK: extracellular regulated protein kinases
JNK: c-Jun N-terminal kinase
AP-1: activator protein-1
SP-1: transcription factor-1
BMP: bone morphogenetic protein
SMAD: mothers against decapentaplegic homolog
R-SMAD: receptor-activated mothers against decapentaplegic homolog
Co-SMAD: common pathway mothers against decapentaplegic homolog
I-SMAD: inhibitory mothers against decapentaplegic homolog
RUNX2: runt-related transcription factor 2
OSX: recombinant osterix
Th: helper T cells
CTL: cytotoxic T lymphocyte
Tregs: regulatory T cells
IL-4: interleukin-4
IL-6: interleukin-6
IL-10: interleukin-10
P2X7: purinergic receptor p2x, ligand gated ion channel, 7
HO: heterotopic ossification
Fra-1: Fos related antigen-1
NK: natural killer
DCs: dendritic cells
IFN-γ: interferon-γ
TNF-β: tumor necrosis factor-β
PGE2: prostaglandin E2
IDO: indoleamine 2,3-dioxygenase
HGF: hepatocyte growth factor
CCL2: C-C motif ligand 2
VEGF: vascular endothelial growth factor
M-CSF: macrophage colony stimulating factor
STAT3: activating signal transducers and activators of transcription 3
